# A Novel Fluorescent Dye Extracted from *Buddleja officinalis* for Labeling Mitochondria after Fixation

**DOI:** 10.1155/2022/7486005

**Published:** 2022-06-03

**Authors:** Ying Yang, Ok-Su Kim, Byunggook Kim, Guo Liu, Jianan Song, Danyang Liu, Guowu Ma, Young Kim, Okjoon Kim

**Affiliations:** ^1^Department of Oral Pathology, School of Dentistry, Chonnam National University, Gwangju 61186, Republic of Korea; ^2^Dental Implant Center, School and Hospital of Stomatology, Wenzhou Medical University, Wenzhou 325000, China; ^3^Department of Periodontology, School of Dentistry, Chonnam National University, Gwangju 61186, Republic of Korea; ^4^Department of Oral Medicine, School of Dentistry, Chonnam National University, Gwangju 61186, Republic of Korea; ^5^Department of Endodontics, School and Hospital of Stomatology, Wenzhou Medical University, Wenzhou 325000, China; ^6^Department of Conservative Dentistry, School of Dentistry, Dental Science Research Institute, Chonnam National University, Gwangju 61186, Republic of Korea; ^7^Department of Oral and Maxillofacial Surgery, School of Stomatology, Dalian Medical University, Dalian 116044, China

## Abstract

Mitochondria are versatile organelles and function by communicating with cellular ecosystems. The fluorescent colocalization analysis after fixation is a highly intuitive method to understand the role of mitochondria. However, there are few fluorescent dyes available for mitochondrial staining after fixation. In this study, a novel fluorescent dye (BO-dye), extracted from *Buddleja officinalis*, was applied for mitochondrial staining in fixed immortalized human oral keratinocytes. The BO-dye (excitation: 414 nm, emission: 677 nm) is a small fluorescent molecular dye, which can cross the cytomembrane without permeabilization. We assume that the BO-dye could aggregate and bind to the mitochondria stably. BO-dye exhibited a mega-Stokes shift (>250 nm), which is an important feature that could reduce self-quenching and enhance the signal-to-noise ratio. Analysis of photophysical properties revealed that the BO-dye is temperature and pH insensitive, and it exhibits superior photostability. These results indicate that BO-dye can be considered an alternative fluorescent dye for labeling mitochondria after fixation.

## 1. Introduction

Mitochondria are vital organelles regarded as the sentinels located at the gate between life and death in the eukaryotic cell [1]. Growing literature has demonstrated that mitochondria are closely associated with various physiological and pathological processes, including energy generation, cell signaling, calcium homeostasis, gene expression, and apoptosis [2–4].

However, the regulation of these two processes does not rely on the mitochondria itself but on the communication between the mitochondria and cellular ecosystem. Several studies have demonstrated that the mitochondria induce intrinsic apoptosis after translocation of apoptosis-related proteins from the cytoplasm to mitochondria [5, 6]. The mitochondria also regulate inflammation by releasing mitochondrial DNA into the cytosol [7, 8]. Furthermore, the crosstalk between the nucleus and mitochondria during mitochondrial dysfunction occurs through the transcription and translocation of genes from the nucleus to mitochondria [9, 10].

To investigate the role of mitochondria in regulating cell fate, various experimental methods have been employed, such as western blotting, real-time quantitative PCR, fluorescent staining, flow cytometry, and enzyme-linked immunosorbent assay. Nonetheless, colocalization analysis based on fluorescent staining after fixation is a highly intuitive method to evaluate the communication between the mitochondria and cell ecosystem, which is attributed to its convenience, simplicity, high selectivity and sensitivity, and noninvasive treatment [11, 12].

So far, the localization analysis of mitochondria after fixation is primarily performed through immunofluorescent staining and fluorescent molecular staining. However, we cannot ignore the disadvantages of these two methods. Immunofluorescent staining is time-consuming, involves painstaking procedures, and results in nonspecific staining [13]. The application of fluorescent molecular staining is limited due to the disadvantages associated with fluorescent molecular dyes, including low cost-effectiveness, poor photophysical stability, and weak signal-to-noise ratio [14]. Altogether, it is necessary to develop an economical and stable fluorescent dye that can be used to label mitochondria rapidly and effectively after fixation.

Natural product libraries are a good source for identifying and developing novel fluorescent dyes. Few fluorescent products have previously been isolated from plants for cellular imaging [15, 16]. In our previous studies, we reported that photosensitizers could be extracted from traditional plants and used for photodynamic treatment [17, 18]. After treating with photosensitizers, fluorescence was identified around the nucleus where mitochondria are located. Thereafter, mitochondria-induced apoptosis was identified after irradiating with red light. Therefore, we hypothesized that these photosensitizers extracted from plants could be used as fluorescent dyes for labeling mitochondria.

In this study, we extracted a fluorescent dye from *Buddleja officinalis* (BO), a source of our previous photosensitizer, and named it as BO-dye. BO-dye was proven to be a stable and cell permeable dye with mega-Stokes shift, which was used for labeling mitochondria after fixation of immortalized human oral keratinocytes (IHOKs).

## 2. Materials and Methods

### 2.1. Extraction of BO-Dye

The flower buds of *B. officinalis* were purchased from Kyung Dong Pharm (Kyung Dong, Korea). The dried herb was ground to obtain a powder using a mechanical grinder (PN, Korea). 200 g of the powder was weighed and suspended in 2000 mL of 70% ethanol. The mixture was stirred with an automatic stirrer (MTOPS, Korea) at 300 rpm for 48 h at 24-26°C. Finally, we obtained a 39.4 g ethanolic extract after filtering and evaporating using a rotary vacuum evaporator (EYELA, Japan). Further, 20 g of ethanolic extract of BO was resuspended in 100 mL of ddH_2_O and sonicated using an ultrasonic cleaner (Bransonic, USA) for 5 min. Then, the suspension liquid was fractionated with 200 mL of *n*-hexane (DUKSAN, Korea), 200 mL of dichloromethane (DUKSAN), 200 mL of ethyl acetate (DUKSAN), and 200 mL of *n*-butanol (DUKSAN) sequentially. Five fractions were filtered and evaporated to yield solid extracts (Figure S1). Each fraction extract was dissolved in dimethyl sulfoxide (DMSO; Sigma-Aldrich, USA) to obtain the same concentration (200 *μ*g/mL). Then, the UV-visible absorption spectrum (300–700 nm) of each extract was measured using a microplate reader (BioTek, USA). Ethyl acetate and dichloromethane extracts exhibited the highest absorption peaks in UV light and visible light, respectively. The fluorescent spectra of these two extracts were recorded using a microplate reader as described below. Absorption peak was first fixed as the excitation wavelength to record the emission spectrum. Then, the emission peak was fixed as the emission wavelength to record the excitation spectrum. The dichloromethane extract was identified as BO-dye as it exhibited the fluorescent spectra similar to that of the photosensitizer from our previous study [17].

### 2.2. Solution of BO-Dye

The stock solution of BO-dye was prepared at a concentration of 10,000 *μ*g/mL in DMSO and stored at -4°C. For photophysical experiments, BO-dye stock was diluted using DMSO unless stated.

### 2.3. Dose-Dependent Stability of BO-Dye

The BO-dye stock was diluted to the indicated concentrations (10, 100, and 500 *μ*g/mL). We then examined the fluorescent spectrum of each sample as described above.

### 2.4. Solvent-Dependent Stability of BO-Dye

The BO-dye stock was diluted in DMSO or phosphate-buffered saline (PBS). The fluorescent spectrum of each sample was then examined as described above.

### 2.5. pH Sensitivity of BO-Dye

We prepared PBS solutions with different pH values (pH 6–9) using 1 M hydrochloric acid (Merck, USA) and 1 M sodium hydroxide (DUKSAN). 10 *μ*L of BO-dye stock was added to 1 mL PBS solution at different pH values. After 48 h of equilibration at 24-26°C, the emission spectrum of each solution was assessed.

### 2.6. Thermosensitivity of BO-Dye

BO-dye stock was diluted to 100 *μ*g/mL, and the solution was equilibrated in a microplate reader at 25, 37, or 45°C for 1 h. We then examined the emission spectrum of each group.

### 2.7. Photostability of BO-Dye

Solutions of BO-dye and MitoView™ Green (Biotium, USA), a commercial mitochondrial staining dye with identical fluorescent intensity (FI), were prepared. Then, 1 mL of each solution was placed in closed metal cuvettes and then illuminated continuously using a blue LED light source (425 nm) with the irradiance of 150 mW/cm^2^. The FI of each sample was recorded every 5 min (excitation/emission of BO-dye: 414 nm/677 nm; MitoView™ Green: 490 nm/523 nm).

### 2.8. Cell Line and Cell Culture

IHOKs were cultured in Dulbecco's modified Eagle medium (DMEM, WELGENE, Korea), supplemented with 10% fetal bovine serum (FBS; Atlas Biologicals, Korea) and 1% penicillin/streptomycin (WELGENE, Korea), and incubated at 37°C in a humid atmosphere with 5% CO_2_. For the staining experiment, we seeded IHOK cells onto the culture slides (SPL, Korea) at a density of 1 × 10^5^ cells/mL and incubated for 24 h before staining.

### 2.9. Staining Condition of BO-Dye

After thorough washing with prewarmed PBS solution, cells were fixed with 4% paraformaldehyde (Tech&Innovation, Korea) for 5 min. The fixed cells were stained with different doses of BO-dye (0.1, 1, 5, and 10 *μ*g/mL) for 10 min. Finally, cells were washed twice with PBS solution and mounted onto the slides using the mounting medium (Sigma-Aldrich). Fluorescent images were acquired using a fluorescence microscope (Lionheart™ FX, USA) with a 445 nm excitation and 685 nm emission filter. After screening the staining dose, we performed time-dependent staining (1, 3, 5, and 10 min) to screen the optimal staining time using the same protocol.

### 2.10. Costaining with Phalloidin, 4′,6-Diamidino-2-phenylindole (DAPI), and BO-Dye

Cells were washed with prewarmed PBS solution twice and fixed with 4% paraformaldehyde at 24-26°C for 15 min. After fixation, cells were washed thrice with PBS solution. Subsequently, fixed cells were treated with 0.1% Triton X-100 (YAKURI, Japan) for 3 min and then washed thrice with PBS solution. To visualize the cytoskeleton, cells were treated with TRTIC-conjugated phalloidin (Millipore, USA) at 24-26°C for 40 min and then washed thrice with PBS solution. Thereafter, cells were treated with BO-dye (1 *μ*g/mL, 3 min) and DAPI (1 : 10000, 3 min). Finally, the images were acquired using a fluorescence microscope after mounting (excitation/emission of BO-dye: 445 nm/685 nm; TRTIC-conjugated phalloidin: 531 nm/593 nm; DAPI: 377 nm/447 nm).

### 2.11. Costaining of BO-Dye and MitoView™ Green

After washing with prewarmed PBS solution, cells were fixed with 4% paraformaldehyde at 24-26°C for 5 min. Following the fixation step, cells were stained with BO-dye and MitoView™ Green, respectively. Finally, cells were mounted onto the slide after washing with PBS solution. Images were acquired using a fluorescence microscope (excitation/emission of BO-dye: 445 nm/685 nm; MitoView™ Green: 469 nm/525 nm). Colocalization of BO-dye and MitoView™ Green was analyzed using the ImageJ software (NIH, USA). We calculated Pearson's correlation coefficient (PCC) and Manders' colocalization coefficient (MCC) above threshold followed by the subtraction of local background (rolling ball radius: 50 pixels). The scatter plot of BO-dye and MitoView™ Green and fluorescence intensity profiles of the linear region of interest (ROIs) were also evaluated.

## 3. Results

### 3.1. Extraction of BO-Dye

**Figure 1:**
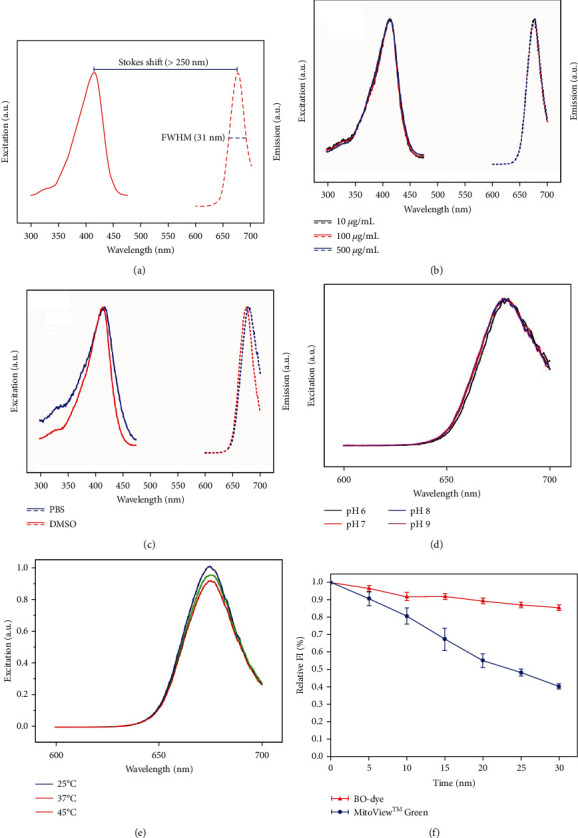
Photophysical properties of BO-dye. (a) The excitation and emission spectra of BO-dye. Excitation/emission: 414 nm/677 nm, full width at half maxima (FWHM): 31 nm. (b) The excitation and emission spectra of BO-dye at various concentrations. (c) The excitation and emission spectrum of BO-dye dissolved in different solvents. (d) The emission spectrum of BO-dye dissolved in phosphate-buffered saline with different pH values (pH 6–9). (e) The emission spectrum of BO-dye at various temperatures. (f) Comparison of photostability of BO-dye with MitoView™ Green.

The BO ethanolic extract was suspended in ddH_2_O and eluted in solvents with increasing polarity (*n*-hexane, dichloromethane, ethyl acetate, and *n*-butanol) to obtain five extracts. Ethyl acetate and dichloromethane extracts exhibited the main absorption peaks in UV and visible lights, respectively (Figure S2a). The emission from the ethyl acetate extract was blue, but that from the dichloromethane extract was red (Figure S2b). The dichloromethane extract was identified as BO-dye because of the similar fluorescence profile as that of our previous photosensitizer [17]. The excitation and emission of BO-dye were 414 nm and 677 nm, respectively, indicating a mega-Stokes shift (>250 nm). Meanwhile, the full width at half maxima (FWAM) of the emission spectral curve was found to be approximately 31 nm (Figure 1(a)).

### 3.2. Stability of BO-Dye

The spectral properties of BO-dye at various concentrations ranging from 10 to 500 *μ*g/mL were examined. The excitation and emission spectra of different groups were almost the same (Figure 1(b)). We further investigated whether the solvent could affect the fluorescence spectrum of BO-dye. The excitation and emission spectra of BO-dye showed a redshift when dissolved in PBS. However, this redshift was short, and the Stokes shift remained large (Figure 1(c)).

The fluorescence of dye might change with different pH or temperatures. In the narrow physical pH range, the emission spectrum was found to be stable against different acidic and alkaline conditions (Figure 1(d)). The fluorescent spectrum of BO-dye at various temperatures was then studied. We found a common emission for each temperature group. Although FI of the peak gradually decreased along with an increase in temperature from 25 to 45°C, the loss was restricted to 10% (Figure 1(e)).

Photostability is one of the most important criteria for developing a novel fluorescent dye. After 30 min of continuous illumination, the emission FI of MitoView™ Green decreased by around 60% when compared to the original, but that of BO-dye only decreased by less than 15% (Figure 1(f)).

### 3.3. Cellular Staining with BO-Dye after Fixation

We explored the possibility of cellular staining with BO-dye after fixation in a dose- and time-dependent manner. BO-dye could cross the cytomembrane easily without the permeabilization of the cytomembrane (Figure S3). The distribution of fluorescence of BO-dye is coincident with that of cells, and the FI increased with an increase in the dose and time. For dose-dependent staining, the fluorescence could be observed in a higher-dose group (≥1 *μ*g/mL), but not a lower-dose group (<1 *μ*g/mL; Figure S3a). For time-dependent staining, shorter time (<3 min) exhibited weaker fluorescence and insufficient staining, but excessive time (>3 min) caused dye diffusion (Figure S3b). Therefore, we concluded that 1 *μ*g/mL and 3 min were optimal staining conditions for further experiments.

### 3.4. Subcellular Localization of BO-Dye

**Figure 2:**
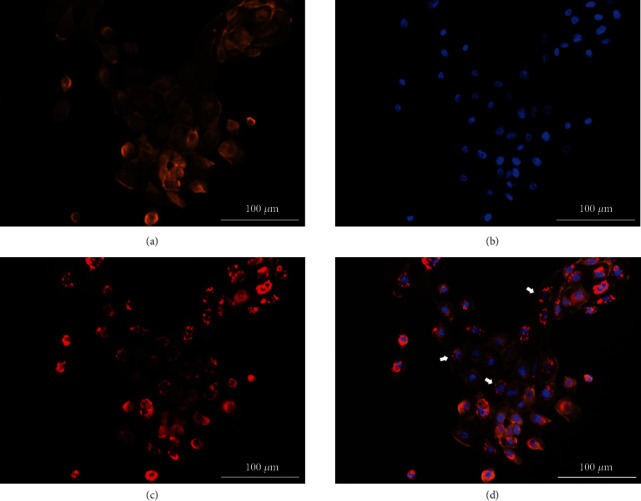
Costaining of phalloidin, 4′,6-diamidino-2-phenylindole (DAPI), and BO-dye in fixed immortalized human oral keratinocytes (IHOKs). (a) Phalloidin channel. (b) DAPI channel. (c) BO-dye channel. (d) Merged channel. BO-dye was distributed around the nucleus but not inside the nucleus and cytomembrane (white arrow). Scale bar is set to 100 *μ*m.

**Figure 3:**
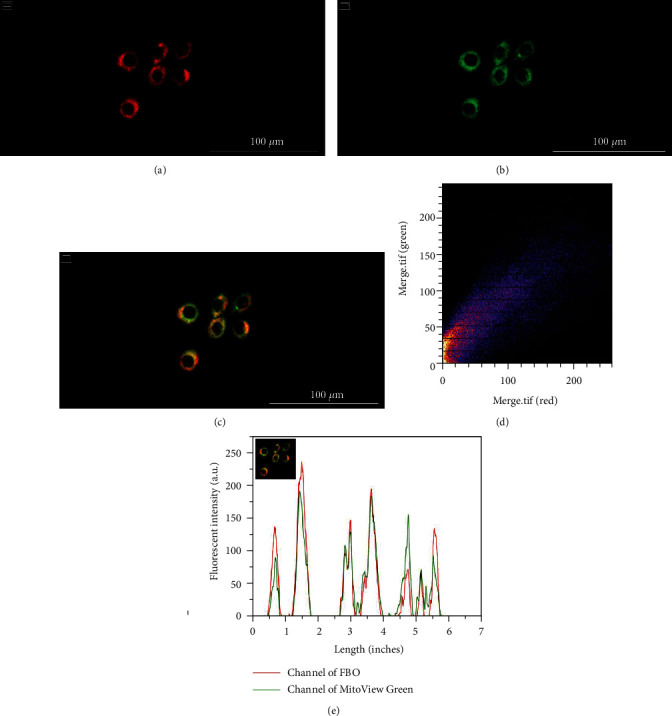
Costaining of BO-dye and MitoView™ Green in fixed IHOKs. (a) BO-dye channel. (b) MitoView™ Green channel. (c) Merged channel. Pearson's correlation coefficient (PCC): 0.93; Manders' colocalization coefficient (MCC): M1 = 0.981, M2 = 0.969. (d) The scatter plot of BO-dye and MitoView™ Green indicated colocalization. (e) Fluorescence intensity profile of the linear region of interest across IHOKs revealed that the fluorescence intensity of BO-dye and MitoView™ Green varies in a close trend. Scale bar is set to 100 *μ*m.

To analyze the distribution of BO-dye, the costaining with BO-dye, phalloidin, and DAPI was performed. The cytoskeleton was visualized with phalloidin staining, and the nucleus was stained with DAPI (Figures 2(a) and 2(b)). After staining with BO-dye, we realized that BO-dye was distributed around the nucleus but not inside the nucleus and cytomembrane (Figures 2(c) and 2(d)). Further, to verify if BO-dye labeled the mitochondria, we costained fixed IHOKs with BO-dye and MitoView™ Green. The fluorescence distribution of both BO-dye and MitoView™ Green was found to be around the nucleus, and their FI were basically the same (Figures 3(a) and 3(b)). According to the merging image (Figure 3(c)), the mathematical analysis of the pixel localization indicated colocalization (PCC: 0.93; M1: 0.981, M2: 0.969). The fluorescent scatter plot also indicated colocalization (Figure 3(d)). The fluorescent intensity profiles of the linear ROIs across IHOKs revealed that the FI of these two dyes vary in a close trend (Figure 3(e)).

## 4. Discussion

In our previous study, the ethanolic extract of BO was found to serve as a photosensitizer [17]. Herein, we further fractionated the photosensitizer using the solvent extraction method to obtain highly purified fluorescent dye. Although several fractions exhibited the fluorescent characteristic, the dichloromethane extract was selected as the lipophilic BO-dye, mainly due to its fluorescence spectrum, which was similar to that of our previous photosensitizer [17].

In this study, BO-dye was confirmed to be an effective tool for labeling mitochondria in fixed cells, because it was easy and quick to use. Many mitochondrial studies have been performed using live cells because the function of mitochondria exhibits a close relationship with its membrane potential [19–24]. However, fixed mitochondrial staining with BO-dye allows for the development of an alternate approach to investigate the role of mitochondria. For example, BO-dye can be used in multiple fluorescence staining to evaluate the communication between mitochondria and other organelles, proteins, or genes.

Cellular staining also revealed that the cytomembrane was permeable to BO-dye, suggesting that the staining with BO-dye depends on the small fluorescent molecules. Compared with the immunofluorescence staining, fluorescent molecular staining could help avoid many limitations, including high cost, time-consuming procedures, and nonspecific binding of the primary and secondary antibodies [13].

We next investigated the mitochondrial labeling pattern of BO-dye. Mitochondrial staining after fixation with fluorescent molecular dyes can be performed through (i) emitting fluorescence after modification by mitochondria or their products [25] and (ii) stably binding with mitochondria [26–28]. In our study, we assume that after reaching the cytosol, BO-dye could aggregate and bind to the mitochondria stably. Since the molecular structure of BO-dye and its staining mechanism have not been elucidated yet, further study is warranted for facilitating our understanding of BO-dye.

Stokes shift is used for describing the energic difference between excitation and emission [29]. A large Stokes shift (≥80 nm) is essential for the application of florescent dye in cellular imaging, which is mainly because it helps in avoiding the overlap between the excitation and emission spectra, reducing self-quenching, and enhancing the signal-to-noise ratio [30–32]. Unfortunately, most of the commercial dyes for mitochondrial staining exhibit a small Stokes shift (≤70 nm) [28, 30]. In this study, the excitation and emission of BO-dye were around 414 nm and 677 nm, respectively, indicating a mega-Stokes shift (>250 nm).

Stability is another important feature of a fluorescent dye that needs to be considered for biological application. Our results showed that the excitation and emission spectrum of BO-dye in DMSO did not change within the range of 10–500 *μ*g/mL, demonstrating that aggregates were not formed. In PBS buffer, a short redshift of the excitation and emission spectrum was observed, which could be because the fluorescent spectra of BO-dye might be changed slightly when dissolved in different polarity solvent. Further study revealed that BO-dye is insensitive to changes in the pH value (pH 6–9) and temperature (25–45°C). This insensitivity would contribute to the stable staining of BO-dye in physiological environment. More importantly, BO-dye exhibited higher photostability when compared with MitoView™ Green, a commercial MitoTracker, which is beneficial for long-term observation, especially under the irradiation of a high-power laser source [33].

In our study, BO-dye also exhibited a narrowband emission with a FWHM of 31 nm, suggesting that this dye emits a bright fluorescence and can be applied in multiple-color staining.

## 5. Conclusion

In summary, our results suggest that BO-dye can be considered an alternate fluorescent dye for labeling the mitochondria after fixation.

## Data Availability

The data used to support the findings of this study are available from the corresponding author upon request.

## Supplementary Materials

Supplementary Materials Figure S1: the BO-dye extraction with various solvents. 39.4 g ethanol extract was obtained from 200 g of dried BO. Approximately 0.26 g of dichloromethane fraction was obtained from 20 g ethanol extract finally. The extraction efficiency was about 1.3%. Figure S2: extraction of BO-dye. (a) UV-visible absorption spectra of five fractions. Ethyl acetate (blue arrow) and dichloromethane extracts (red arrow) exhibited the main absorption peaks in UV and visible lights, respectively. (b) The emission from the ethyl acetate extract was blue, but that from the dichloromethane extract was red. Solid line: excitation spectrum; short dash line: emission spectrum. Figure S3: the dose- and time-dependent staining of BO-dye in fixed immortalized human oral keratinocytes (IHOKs). (a) Dose-dependent staining. The fluorescence could be observed in a higher-dose group (≥1 *μ*g/mL), but not a lower-dose group (<1 *μ*g/mL). (b) Time-dependent staining. Shorter time (<3 min) revealed weaker fluorescence and insufficient staining, but excessive time (>3 min) caused dye diffusion. Excitation/emission of BO-dye: 445 nm/685 nm. Scale bar is set to 200 *μ*m.
