# Supplementary material for: A Novel Fluorescent Dye Extracted from Buddleja officinalis for Labeling Mitochondria after Fixation
Source: Scanning. 2022 Jun 3;2022:7486005. doi: 10.1155/2022/7486005 (PMC9187463; doi:10.1155/2022/7486005)
Supplement: Supplementary Materials — Figure S1: the BO-dye extraction with various solvents. 39.4 g ethanol extract was obtained from 200 g of dried BO. Approximately 0.26 g of dichloromethane fraction was obtained from 20 g ethanol extract finally. The extraction efficiency was about 1.3%. Figure S2: extraction of BO-dye. (a) UV-visible absorption spectra of five fractions. Ethyl acetate (blue arrow) and dichloromethane extracts (red arrow) exhibited the main absorption peaks in UV and visible lights, respectively. (b) The emission from the ethyl acetate extract was blue, but that from the dichloromethane extract was red. Solid line: excitation spectrum; short dash line: emission spectrum. Figure S3: the dose- and time-dependent staining of BO-dye in fixed immortalized human oral keratinocytes (IHOKs). (a) Dose-dependent staining. The fluorescence could be observed in a higher-dose group (≥1 μg/mL), but not a lower-dose group (<1 μg/mL). (b) Time-dependent staining. Shorter time (<3 min) revealed weaker fluorescence and insufficient staining, but excessive time (>3 min) caused dye diffusion. Excitation/emission of BO-dye: 445 nm/685 nm. Scale bar is set to 200 μm. [file 7486005.f1.docx]

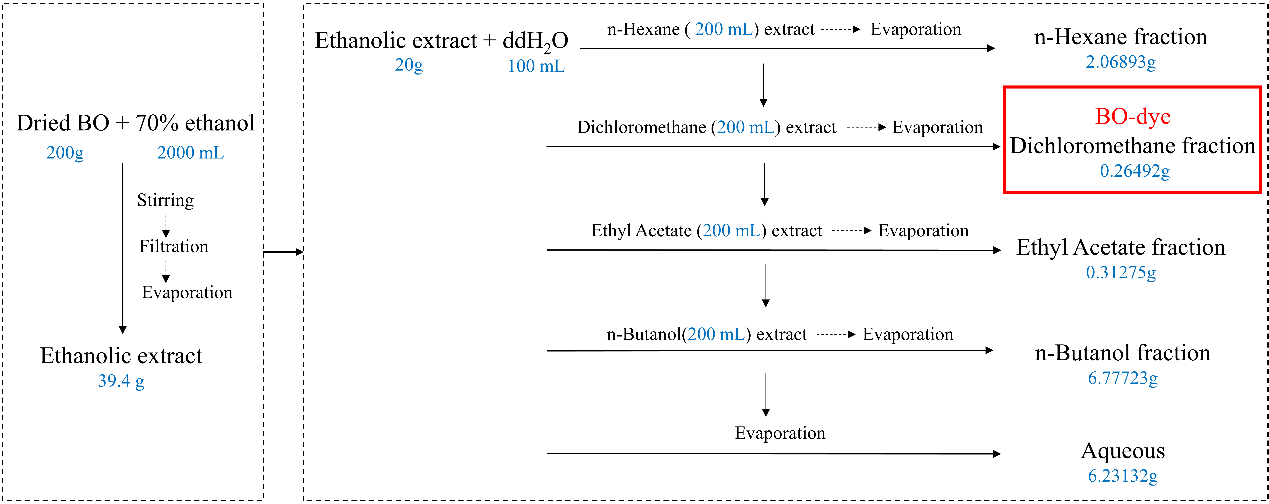


**FIGURE S1.** The BO-dye extraction with various solvents. 39.4 g ethanol extract was obtained from 200 g of dried BO. Approximately 0.26 g of dichloromethane fraction was obtained from 20g ethanol extract finally. The extraction efficiency was about 1.3%.

**
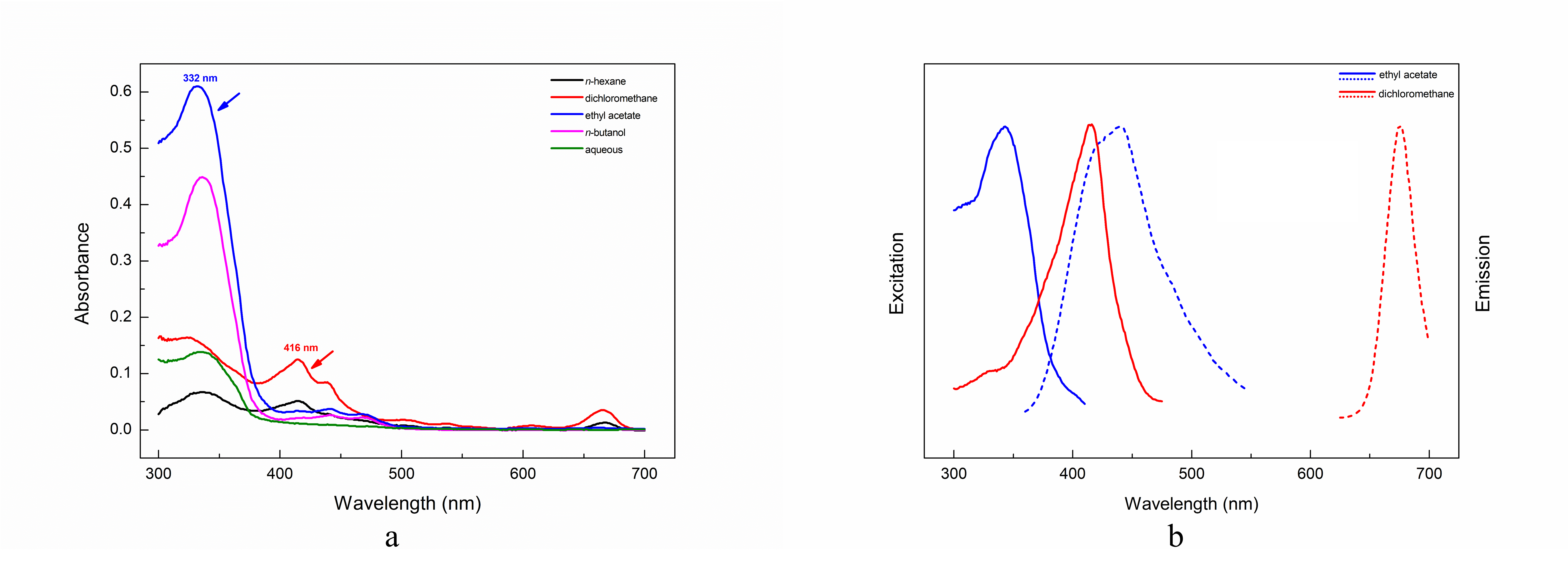
**

**FIGURE S2.** Extraction of BO-dye (a) UV-Visible absorption spectra of five fractions. Ethyl acetate (blue arrow) and dichloromethane extracts (red arrow) exhibited the main absorption peaks in UV and visible lights, respectively. (b) The emission from ethyl acetate extract was blue, but that from dichloromethane extract was red. solid line: excitation spectrum; short dash line: emission spectrum.

**
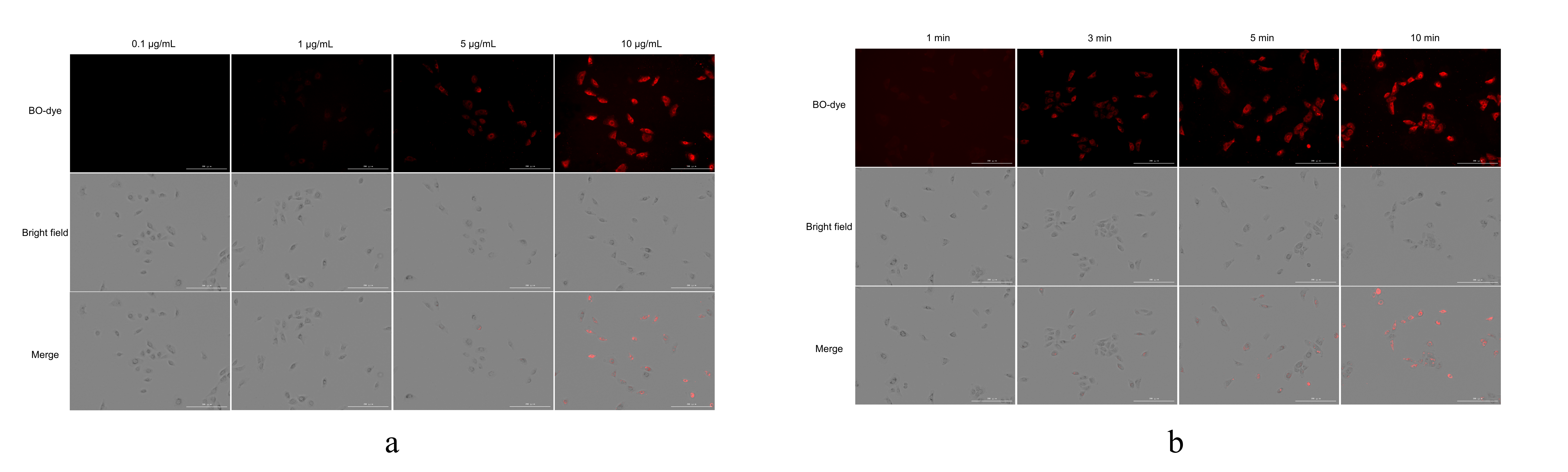
**

**FIGURE S3.** The dose- and time-dependent staining of BO-dye in fixed Immortalized human oral keratinocytes (IHOKs). (a) Dose-dependent staining. The fluorescence could be observed in higher dose group (≥1 µg/mL), but not lower dose group (< 1 µg/mL) (b) Time-dependent staining. Shorter time (< 3 min) revealed weaker fluorescence and insufficient staining, but excessive time (> 3 min) caused dye diffusion. Excitation/emission of BO-dye: 445 nm/685 nm. Scale bar is set to 200 µm.
